# Application of comparative genomics of *Acetobacter* species facilitates genome-scale metabolic reconstruction of the *Acetobacter ghanensis* LMG 23848^T^ and *Acetobacter senegalensis* 108B cocoa strains

**DOI:** 10.3389/fmicb.2022.1060160

**Published:** 2022-11-24

**Authors:** Rudy Pelicaen, Stefan Weckx, Didier Gonze, Luc De Vuyst

**Affiliations:** ^1^Research Group of Industrial Microbiology and Food Biotechnology, Faculty of Sciences and Bioengineering Sciences, Vrije Universiteit Brussel, Brussels, Belgium; ^2^ULB-VUB Interuniversity Institute of Bioinformatics in Brussels, Brussels, Belgium; ^3^Unité de Chronobiologie Théorique, Service de Chimie Physique, Faculté des Sciences, Université libre de Bruxelles, Brussels, Belgium

**Keywords:** cocoa fermentation, *Acetobacter* metabolism, comparative genomics, genome-scale metabolic modelling, flux balance analysis

## Abstract

*Acetobacter* species play an import role during cocoa fermentation. However, *Acetobacter ghanensis* and *Acetobacter senegalensis* are outcompeted during fermentation of the cocoa pulp-bean mass, whereas *Acetobacter pasteurianus* prevails. In this paper, an *in silico* approach aimed at delivering some insights into the possible metabolic adaptations of *A. ghanensis* LMG 23848^T^ and *A. senegalensis* 108B, two candidate starter culture strains for cocoa fermentation processes, by reconstructing genome-scale metabolic models (GEMs). Therefore, genome sequence data of a selection of strains of *Acetobacter* species were used to perform a comparative genomic analysis. Combining the predicted orthologous groups of protein-encoding genes from the *Acetobacter* genomes with gene-reaction rules of GEMs from two reference bacteria, namely a previously manually curated model of *A. pasteurianus* 386B (iAp386B454) and two manually curated models of *Escherichia coli* (EcoCyc and iJO1366), allowed to predict the set of reactions present in *A. ghanensis* LMG 23848^T^ and *A. senegalensis* 108B. The predicted metabolic network was manually curated using genome re-annotation data, followed by the reconstruction of species-specific GEMs. This approach additionally revealed possible differences concerning the carbon core metabolism and redox metabolism among *Acetobacter* species, pointing to a hitherto unexplored metabolic diversity. More specifically, the presence or absence of reactions related to citrate catabolism and the glyoxylate cycle for assimilation of C2 compounds provided not only new insights into cocoa fermentation but also interesting guidelines for future research. In general, the *A. ghanensis* LMG 23848^T^ and *A. senegalensis* 108B GEMs, reconstructed in a semi-automated way, provided a proof-of-concept toward accelerated formation of GEMs of candidate functional starter cultures for food fermentation processes.

## Introduction

Acetic acid bacteria (AAB) are Gram-negative, obligately aerobic bacteria that incompletely oxidize a range of alcohols and reducing sugars for energy production. The region-and stereo-selectivity of these oxidation reactions has led to numerous biotechnological applications, most notably the conversion of *D*-sorbitol into *L*-sorbose (Shinjoh and Toyama, 2016). The genus *Acetobacter* comprises tens of species and is common to food fermentation processes, among which traditional vinegar production, kombucha fermentation, and cocoa fermentation (De Vuyst and Weckx, 2016; Yamada, 2016; Coton et al., 2017; Lu et al., 2018; De Vuyst and Leroy, 2020). For the latter fermentation process, several *Acetobacter* species have been isolated and selected as candidate functional starter cultures, including *Acetobacter ghanensis* LMG 23848^T^ and *Acetobacter senegalensis* 108B (Camu et al., 2007; Moens et al., 2014; Illeghems et al., 2016). To gain more insights into their contribution to cocoa fermentation processes, their genomes have been sequenced and functionally annotated, revealing a possible role in citrate assimilation during fermentation of the cocoa pulp-bean mass (Illeghems et al., 2016). However, *A. ghanensis* and *A. senegalensis* are outcompeted during cocoa fermentation, whereas *Acetobacter pasteurianus* prevails (Lefeber et al., 2010, 2011, 2012; Papalexandratou et al., 2013; Díaz-Muñoz et al., 2021).

The gold standard in the taxonomic analysis of AAB are polyphasic approaches, combining phenotypic, chemotaxonomic, and genotypic data (Cleenwerck and De Vos, 2008; De Vuyst et al., 2008; Papalexandratou et al., 2009; Li et al., 2017). One specific metabolic feature that is typically tested for differentiating *Acetobacter* species is growth on ethanol as the sole carbon source. Therefore, a defined medium containing ethanol as the sole carbon source and ammonium as the sole nitrogen source is often used (Cleenwerck et al., 2002). However, carbon source usage may also be deduced through genomic analysis, since carbon assimilation pathways consist of a defined sequence of biochemical reactions, for which the respective enzyme-encoding genes must be present in the genome (Serres and Riley, 2006).

Growth of *Acetobacter* species on C2 compounds, such as ethanol and acetate, has so far been related to the presence of the glyoxylate cycle enzymes (Saeki et al., 1997; Sakurai et al., 2013). The glyoxylate cycle is a shunt of the tricarboxylic acid (TCA) cycle and involves only two enzymes, namely isocitrate lyase (EC 4.1.3.1), which converts isocitrate into glyoxylate and succinate, and malate synthase (EC 2.3.3.9), which binds acetyl-CoA onto glyoxylate to form malate. The glyoxylate cycle circumvents the carbon dioxide-producing reactions of the TCA cycle, thus leading to a net succinate production. The presence of genes encoding the glyoxylate cycle enzymes in *Acetobacter* species genomes and their relationship to the growth of these species in a medium containing ethanol as the sole carbon source has not been thoroughly investigated.

A genome-scale metabolic model (GEM) provides a well-defined data structure of the metabolic network inferred from a bacterial genome (Thiele and Palsson, 2010). Next to the use of a GEM as knowledge base of the biochemical reaction potential of a microbial strain, a GEM is also useful to perform simulations of the metabolism of a microbial strain, for instance using constraint-based modeling methods. GEMs have been used to compare the reaction potential of different microorganisms, and led to the concept of a pan-reactome (Prigent et al., 2018; Seif et al., 2018; Gu et al., 2019). In this case, the GEM reconstruction process is performed for different strains in parallel using different metabolic resources, for instance GEMs of reference species and metabolic databases.

In the present study, the approach mentioned above was applied to reconstruct GEMs for *A. ghanensis* LMG 23848^T^ and *A. senegalensis* 108B, taking advantage of a comparative genomic analysis that encompassed a selection of 36 strains belonging to 27 species of the *Acetobacter* genus and a previously reconstructed and manually curated GEM of *A. pasteurianus* 386B (Pelicaen et al., 2019, 2020). These GEMs will be useful to gain more insights into the metabolism and hence metabolic adaptations of *A. ghanensis* LMG 23848^T^ and *A. senegalensis* 108B to cocoa fermentation conditions by applying *in silico* flux balance analysis.

## Materials and methods

### Strains

Three strains of *Acetobacter* species, originating from spontaneous cocoa fermentation processes carried out in heaps in Ghana in 2004, namely *A. pasteurianus* 386B, *A. ghanensis* LMG 23848^T^, and *A. senegalensis* 108B, were used throughout this study (Camu et al., 2007; Illeghems et al., 2013, 2016; Moens et al., 2014).

### Growth experiments

Growth experiments were performed in a modified defined medium (Verduyn et al., 1992). This medium contained: (NH_4_)_2_SO_4_, 5.0 g/l; KH_2_PO_4_, 1.375 g/l; MgSO_4_.7H_2_O, 0.5 g/l; ethylenediaminetetraacetic acid (EDTA), 15.0 mg/l; ZnSO_4_.7H_2_O, 4.5 mg/l; CoSO_4_.7H_2_O, 0.35 mg/l; MnCl_2_.4H_2_O, 1.0 mg/l; CuSO_4_.5H_2_O, 0.3 mg/l; CaCl_2_.2H_2_O, 4.5 mg/l; FeSO_4_.7H_2_O, 3.0 mg/l; MoO_3_, 0.24 mg/l; H_3_BO_3_, 1.0 mg/l; and KI, 0.1 mg/l, dissolved in 30 mM citrate buffer (pH 6.0). The final pH of the medium was adjusted to 5.0 with 0.1 M NaOH. A filter-sterilised vitamin mixture was added after heat sterilisation of the medium at 121°C for 20 min. The final vitamin concentrations were: biotin, 0.0005 mg/l; calcium pantothenate, 0.01 mg/l; nicotinic acid, 0.01 mg/l; *myo*-inositol, 0.25 mg/l; thiamine-HCl, 0.01 mg/l; pyridoxine-HCl, 0.01 mg/l; and *para*-aminobenzoic acid, 0.002 mg/l. Eight different carbon sources, namely glucose, fructose, mannitol, citric acid, glycerol, lactic acid, ethanol, and acetic acid (as sodium acetate), were used at a final concentration of 30 mM to assess if they could sustain growth of the three *Acetobacter* strains investigated. The pH of the citric acid, lactic acid, and sodium acetate stock solutions was set to 5.0.

For inoculum build-up, the three *Acetobacter* strains were grown overnight at 30°C in a standard incubator (160 rpm) in a modified yeast extract-glucose-mannitol (mYGM) medium (Lefeber et al., 2012), which contained: *D*-glucose, 20.0 g/l; *D*-mannitol, 20.0 g/l; lactic acid, 10.0 g/l; granulated yeast extract, 10.0 g/l; soy peptone, 5.0 g/l; MgSO_4_.7H_2_O, 1.0 g/l; (NH_4_)_2_HPO_4_, 1.0 g/l; KH_2_PO_4_, 1.0 g/l; and Na_3_C_6_H_5_O_7_, 1.0 g/l. The final pH of the medium was adjusted to 5.5 with 0.1 M NaOH. The overnight culture was centrifuged (4,000 x *g*, 20 min, 4°C) and washed with a filter-sterilized saline solution (0.85%, m/v, NaCl). Then, the cells were resuspended in sterile saline solution and inoculated in 2 ml of the defined medium mentioned above at an optical density at 600 nm (OD_600_) of 0.01, in duplicate, in test tubes with a total volume of 20 ml. The three *Acetobacter* strains were allowed to grow at 30°C for 48 h and 120 h. A threshold value for the OD_600_ of 0.1 was used to identify whether or not the strains had grown.

A similar growth experiment was performed in a modified defined medium with 30 mM phosphate buffer (pH 6.0) in triplicate. In this case, for inoculum build-up, the three *Acetobacter* strains were grown overnight at 30°C in a semi-defined medium (pH 5.5), which contained: lactic acid, 5.0 g/l; sodium acetate, 10.0 g/l; granulated yeast extract, 5.0 g/l; MgSO_4_.7H_2_O, 1.0 g/l; NH_4_H_2_PO_4_, 20.0 g/l; and K_2_HPO_4_, 10.0 g/l (Pelicaen et al., 2019). A threshold value for the OD_600_ of 0.1 was used to identify whether or not the strains had grown.

### Re-annotation of the *Acetobacter ghanensis* LMG 23848^T^ and *Acetobacter senegalensis* 108B genomes

The protein-encoding genes of the *A. ghanensis* LMG 23848^T^ and *A. senegalensis* 108B genomes were originally annotated using the bacterial genome annotation system GenDB v2.2 (Meyer, 2003; Illeghems et al., 2016). In the present study, these genomes were re-annotated using a previously in-house developed bioinformatics workflow (Pelicaen et al., 2019). The National Center for Biotechnology Information (NCBI, Bethesda, Madison, United States) RefSeq genome annotation version was taken as a basis for re-annotation of the genome of *A. ghanensis* LMG 23848^T^ (accessed in April 2017; 2,454 protein-encoding genes) and *A. senegalensis* 108B (accessed in October 2017; 3,444 protein-encoding genes). The amino acid sequences of each set of protein-encoding genes were annotated using a combination of tools, namely BlastKOALA from the KEGG database (Kanehisa et al., 2016, 2017), the TransAAP tool to predict transport proteins (Elbourne et al., 2017), the subcellular localization predictor CELLO (Yu et al., 2004), eggNOG-mapper (Huerta-Cepas et al., 2017), the enzyme annotation tool PRIAM (Claudel-Renard et al., 2003), the RAST annotation pipeline from the KBase software (Overbeek et al., 2014; Arkin et al., 2018), and the tools embedded in InterProScan 5.22–61.0 (Jones et al., 2014). Furthermore, since the publication of the genome sequences of *A. ghanensis* LMG 23848^T^ and *A. senegalensis* 108B (Illeghems et al., 2016), these genomes have been re-annotated by several annotation pipelines and the functional annotation data known at the time of analysis were stored in dedicated databases related to those pipelines. As such, these additional functional annotations were retrieved from the Carbohydrate-Active enZYmes (CAZy) database (Lombard et al., 2014), the Pathosystems Resource Integration Center (PATRIC) database (Wattam et al., 2017), and the Universal Protein Resource (UniProt) database (The UniProt Consortium, 2019).

In addition, the genome annotation pipelines used by GenDB, NCBI RefSeq (NCBI prokaryotic genome annotation pipeline; Tatusova et al., 2016) and PATRIC (RASTtk; Brettin et al., 2015) represent independent methods combining gene prediction and gene annotation. Therefore, functional annotation data of these sources were combined in an in-house MySQL database specific for *A. ghanensis* LMG 23848^T^ and *A. senegalensis* 108B.

### Comparative genomics of *Acetobacter* species based on orthogroups

From all *Acetobacter* species mentioned in the NCBI Taxonomy database (accessed May 2019), the genome sequence of 96 strains of *Acetobacter* species was available in the NCBI RefSeq database. All those genome sequences were retrieved, as well as the genome sequences of *Gluconobacter oxydans* 621H, *Komagataeibacter nataicola* RZS01, and *Escherichia coli* str. K-12 substr. MG1655 (further referred to as *E. coli*). The latter three strains were used as outgroup. Next, the *Acetobacter* genomes were ranked according to their assembly quality level, based on information in the NCBI RefSeq database. For each *Acetobacter* species, the genome with the highest assembly quality level was selected, and in the case that the genome was not from the type strain, the type strain genome was also selected. Next, OrthoFinder (Emms and Kelly, 2015) was used to predict orthogroups from the protein-encoding genes of the selected *Acetobacter* genomes and from the three outgroup genomes. Protein identifiers from the NCBI RefSeq database were linked to gene identifiers based on the Genbank files of each genome. Subsequently, OrthoFinder was used to infer a rooted species tree based on the predicted orthogroups (Emms and Kelly, 2017, 2018).

### Reconstruction of genome-scale metabolic models for *Acetobacter ghanensis* LMG 23848^T^ and *Acetobacter senegalensis* 108B

The relationship between genes, proteins, and reactions can be described using gene-protein-reaction (GPR) associations, linking the different entities, possibly with their stoichiometry (Thiele and Palsson, 2010). However, in practice, GPR associations are typically implemented as Boolean rules that define reaction presence based on gene presence in an annotated genome (Machado et al., 2016). Albeit potentially oversimplifying the actual GPR associations, these Boolean rules (gene-reaction rules) allow to quickly evaluate the presence of a reaction in the GEM through *in silico* gene deletions (Ebrahim et al., 2013). In the present study, this concept was exploited to predict the reaction presence based on gene presence in the annotated genomes. To convert the text Boolean expression into a symbolic rule that allows its computational assessment, the Python library SymPy was used (Meurer et al., 2017).

As a first step in the reconstruction of the GEMs for *A. ghanensis* LMG 23848^T^ and *A. senegalensis* 108B, the manually curated GEM of *A. pasteurianus* 386B (iAp386B454; Pelicaen et al., 2019) was used as a reference to evaluate which of the reactions of this GEM were present in the *Acetobacter* genomes considered. To minimize the number of false negative predictions of reaction presence, a separate analysis was performed, whereby only high-quality *Acetobacter* genomes with an NCBI assembly level corresponding to a complete genome were considered.

Next, the predictions obtained were used for the reconstruction of GEMs of *A. ghanensis* LMG 23848^T^, further referred to as iAg23848, and of *A. senegalensis* 108B, further referred to as iAs108B. New gene-reaction rules were associated to the transferred iAp386B454 reactions. For multi-copy orthogroups, i.e., orthogroups containing multiple genes for each genome, the logic ‘OR’ assumption was made, expressing that these genes are co-orthologs of the *A. pasteurianus* 386B genes. Cases for which *A. pasteurianus* 386B genes occurred in the same orthogroup as the ones of *A. ghanensis* LMG 23848^T^ and *A. senegalensis* 108B and had a logic ‘AND’ relation in the iAp386B454 gene-reaction rules, were systematically checked and manually curated in the iAg23848 and iAs108B GEMs. Reactions without GPR, including spontaneous reactions as well as reactions for macromolecular biosynthesis and the iAp386B454 biomass reaction, were transferred as such to the iAg23848 and iAs108B GEMs.

### Gap filling of genome-scale metabolic models and flux balance analysis

Further manual curation was performed for the iAg23848 and iAs108B GEMs in a semi-automated way. First, GEM gap filling was performed using iAp386B454 as a template. Hereto, the GEM gap filling method of CobraPy was used (Ebrahim et al., 2013), which computes the minimal amount of reactions that need to be added from the iAp386B454 template to obtain *in silico* growth. Then, gap filling results were manually curated by combining functional annotation information of *A. ghanensis* LMG 23848^T^ and *A. senegalensis* 108B, stored in their respective MySQL databases and in the GEMs. Only the reactions for which genome annotation evidence was found were added to the GEMs. This also included reactions necessary to reconcile *in silico* and *in vitro* growth experiments. Finally, flux balance analysis (FBA) and parsimonious FBA with biomass formation as the objective function were performed to examine the flux distributions of the newly constructed GEMs under simulated *in vitro* growth conditions. The total allowable carbon consumption flux was set to 60 c-mmol per gram of cell dry mass per h.

### Presence of the glyoxylate cycle and aerobic respiratory chain reactions

To predict the presence/absence of reactions of the glyoxylate cycle and the aerobic respiratory chain, a similar analysis as described in Section 2.4 was performed but now with an *E. coli* GEM as reference. For *E. coli*, two curated reconstruction sources were leveraged, namely the EcoCyc Pathway/Genome Database embedded in Pathway Tools version 21.5, and the iJO1366 GEM of the BIGG database (Orth et al., 2011; Keseler et al., 2017).

## Results

### Growth experiments

Growth experiments (Table 1) under defined medium conditions, using a citrate buffer, indicated that *A. pasteurianus* 386B showed the same growth phenotype as in defined medium with a phosphate buffer. Only the addition of lactic acid as a sole carbon source could sustain the growth of *A. pasteurianus* 386B under these conditions. For *A. ghanensis* LMG 23848^T^, increasing the incubation time from 48 h to 120 h allowed for growth of the bacterial population only on glucose as the sole carbon source, using a citrate buffer. In contrast, the growth phenotype of *A. senegalensis* 108B markedly differed when using a phosphate buffer or citrate buffer. Under growth conditions with a phosphate buffer, this strain only showed growth on lactic acid and citric acid as the sole carbon sources, but exchanging the phosphate buffer for a citrate buffer led to growth on all single carbon sources tested, at least after 120 h of incubation. These results indicated the influence of the medium buffer used, and the possibility of citrate co-consumption in the case that a citrate buffer was applied.

**Table 1:** Indication of bacterial population growth of strains of three different *Acetobacter* species in a modified defined medium with phosphate buffer or citrate buffer, supplemented with different carbon sources, after 48 h and 48 and 120 h of incubation, respectively.

Carbon source	*Acetobacter pasteurianus* 386B		*Acetobacter ghanensis* LMG 23848^T^		*Acetobacter senegalensis* 108B	
	Phosphate 48 h	Citrate 48 h / 120 h	Phosphate 48 h	Citrate 48 h / 120 h	Phosphate 48 h	Citrate 48 h / 120 h
Glucose	−	− / −	−	− / +	−	+ / +
Fructose	−	− / −	−	− / −	−	+ / +
Mannitol	−	− / −	−	− / −	−	+ / +
Citric acid	−	− / −	−	− / −	+	− / +
Glycerol	−	− / −	−	− / −	−	+ / +
Lactic acid	+	+ / +	−	− / −	+	+ / +
Ethanol	−	− / −	−	− / −	−	+ / +
Acetic acid	−	− / −	−	− / −	−	+ / +

−, no growth; +, growth

### Re-annotation of the *Acetobacter ghanensis* LMG 23848^T^ and *Acetobacter senegalensis* 108B genomes

Functional annotation information of *A. ghanensis* LMG 23848^T^ and *A. senegalensis* 108B was stored in their respective MySQL databases. For *A. ghanensis* LMG 23848^T^, it comprised the functional annotations of 2,698 protein-encoding genes originally annotated with GenDB, extended with 160 unique protein-encoding genes from NCBI RefSeq, and 306 unique protein-encoding genes from PATRIC. For *A. senegalensis* 108B, it comprised the functional annotations of 3,605 protein-encoding genes originally annotated with GenDB, extended with 340 unique protein-encoding genes from NCBI RefSeq, and 625 unique protein-encoding genes from PATRIC.

### Comparative genomics of *Acetobacter* species based on orthogroups

The genome selection procedure resulted in 36 genomes (Table 2). Sixteen of these genomes had only a contig level quality, of which eight were type strain genomes. Based on the protein-encoding genes in these genomes, OrthoFinder predicted the presence of 6,149 orthogroups, of which 155 were orthogroups that contained at least one protein-encoding gene for each genome considered, and 98 of these orthogroups contained exactly one protein-encoding gene for each genome (so-called single-copy orthogroups).

**Table 2:** Selection of *Acetobacter* genomes used in the OrthoFinder analysis.

Strain of *Acetobacter* species	NCBI assembly level	NCBI RefSeq identifier
*Acetobacter aceti* TMW2.1153	Complete genome	GCF_002005445.1_ASM200544v1
*Acetobacter aceti* NBRC 14818^T^	Contig	GCF_004341595.1_ASM434159v1
*Acetobacter ascendens* LMG 1590^T^	Complete genome	GCF_001766235.1_ASM176623v1
*Acetobacter cerevisiae* LMG 1625^T^	Contig	GCF_001580535.1_ASM158053v1
*Acetobacter cerevisiae* LMG 1545	Contig	GCF_001581105.1_ASM158110v1
*Acetobacter cibinongensis* 4H-1^T^	Contig	GCF_000963925.1_ASM96392v1
*Acetobacter fabarum* KR	Contig	GCF_002276555.1_ASM227655v1
*Acetobacter ghanensis* LMG 23848^T^	Chromosome	GCF_001499675.1_Acetobacter_ghanensis
*Acetobacter indonesiensis* 5H-1^T^	Scaffold	GCF_000963945.1_ASM96394v1
*Acetobacter malorum* CECT 7742	Scaffold	GCF_001642635.1_ASM164263v1
*Acetobacter malorum* LMG 1746^T^	Contig	GCF_001580615.1_ASM158061v1
*Acetobacter nitrogenifigens* DSM 23921^T^	Scaffold	GCF_000429165.1_ASM42916v1
*Acetobacter okinawensis* JCM 25146^T^	Contig	GCF_000613865.1_ASM61386v1
*Acetobacter orientalis* 21F-2^T^	Scaffold	GCF_000963965.1_ASM96396v1
*Acetobacter orleanensis* CCM 3610	Scaffold	GCF_002358055.1_ASM235805v1
*Acetobacter orleanensis* JCM 7639^T^	Scaffold	GCF_000964205.1_ASM96420v1
*Acetobacter oryzifermentans* SLV-7^T^	Complete genome	GCF_001628715.1_ASM162871v1
*Acetobacter papayae* JCM 25143^T^	Contig	GCF_000613285.1_ASM61328v1
*Acetobacter pasteurianus* Ab3	Complete genome	GCF_001183745.1_ASM118374v1
*Acetobacter pasteurianus* 386B	Complete genome	GCF_000723785.2_AP1
*Acetobacter pasteurianus* subsp. *pasteurianus* LMG 1262^T^	Scaffold	GCF_000285275.1_ASM28527v1
*Acetobacter persici* TMW2.1084	Complete genome	GCF_002006565.1_ASM200656v1
*Acetobacter persici* JCM 25330^T^	Contig	GCF_000613905.1_ASM61390v1
*Acetobacter pomorum* BDGP5	Complete genome	GCF_002456135.1_ASM245613v1
*Acetobacter senegalensis* 108B	Complete genome	GCF_001499615.1_Acetobacter_senegalensis_108B
*Acetobacter senegalensis* LMG 23690^T^	Contig	GCF_001580995.1_ASM158099v1
*Acetobacter* sp. B6	Contig	GCF_004014775.1_ASM401477v1
*Acetobacter* sp. BCRC 14118	Contig	GCF_003332175.1_ASM333217v1
*Acetobacter* sp. DmW_043	Contig	GCF_002153485.1_ASM215348v1
*Acetobacter* sp. DsW_059	Contig	GCF_002153695.1_ASM215369v1
*Acetobacter* sp. DsW_063	Contig	GCF_002153745.1_ASM215374v1
*Acetobacter* sp. DsW_54	Contig	GCF_002153575.1_ASM215357v1
*Acetobacter* sp. JWB	Complete genome	GCF_003323795.1_ASM332379v1
*Acetobacter syzygii* 9H-2^T^	Scaffold	GCF_000964225.1_ASM96422v1
*Acetobacter tropicalis* NBRC 16470^T^	Scaffold	GCF_000787635.2_ASM78763v2
*Acetobacter tropicalis* BDGP1	Complete genome	GCF_002549835.1_ASM254983v1

**Figure 1:**
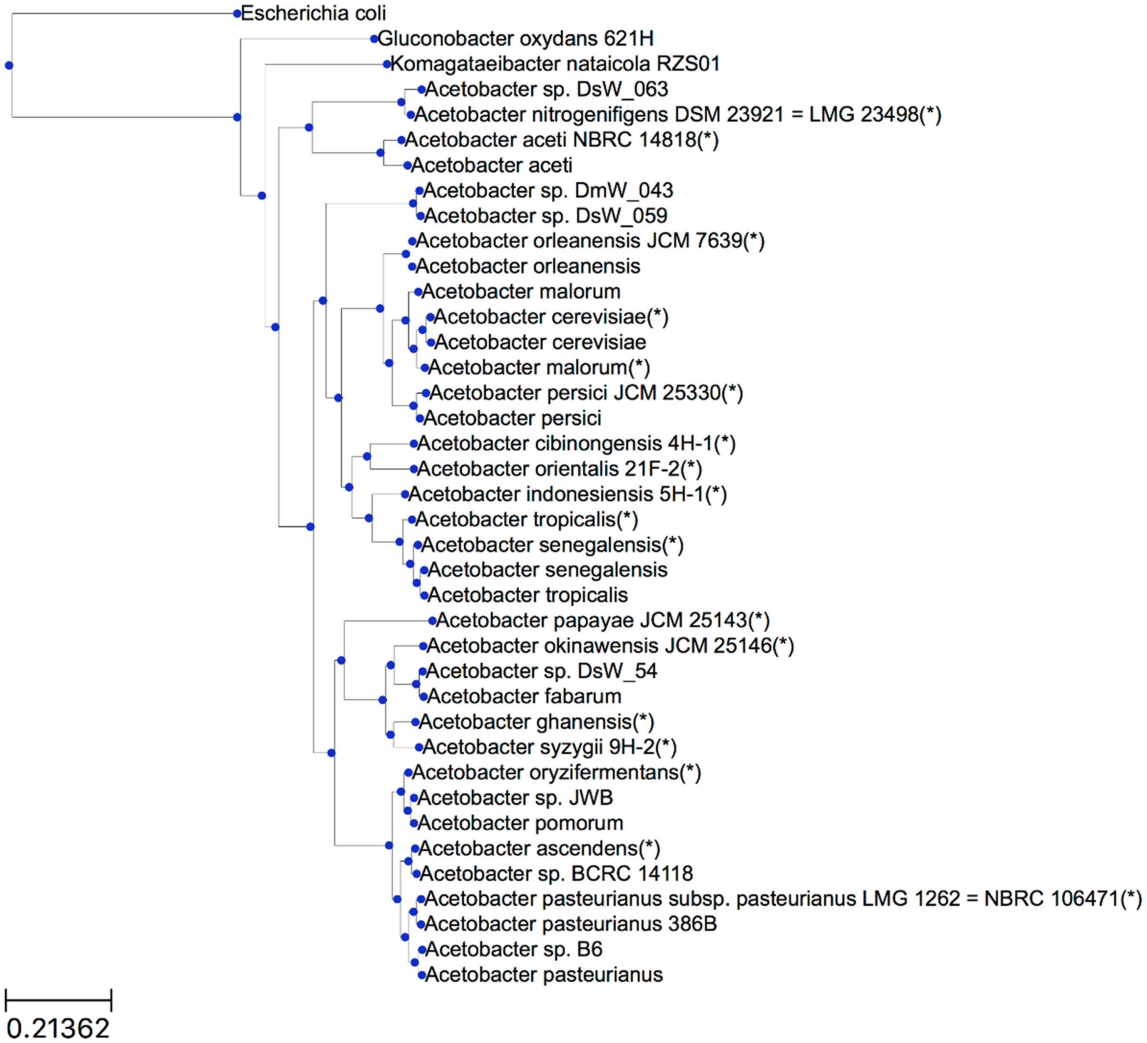
Rooted species tree inference by OrthoFinder. Branch lengths represent the average number of substitutions per site. Type strain genomes are indicated with an asterisk (*). For genomes for which the strain name is not indicated, the reader is referred to Table 2.

Based on all predicted orthogroups, OrthoFinder inferred a rooted species tree (Figure 1). The outgroup genomes chosen, namely *E. coli*, *G. oxydans* 621H, and *K. nataicola* RZS01, were successfully placed outside the *Acetobacter* species subtree by OrthoFinder. Moreover, the tree locations of genomes of hereto unidentified *Acetobacter* species provided a first indication to which *Acetobacter* species they were most related to. It concerned *Acetobacter* sp. DsW_063 (isolated from a fruit fly) as *A. nitrogenifigens*, *Acetobacter* sp. DsW_54 (fruit fly) as *A. fabarum*, *Acetobacter* sp. JWB (fruit fly) as *A. pomorum*, *Acetobacter* sp. BCRC 14118 (African vinegar) as *A. ascendens* (formerly identified as *A. pasteurianus*), and *Acetobacter* sp. B6 (South Korean vinegar) as *A. pasteurianus*.

### Reconstruction of genome-scale metabolic models for *Acetobacter ghanensis* LMG 23848^T^ and *Acetobacter senegalensis* 108B

The predicted orthogroups were used in combination with the *A. pasteurianus* iAp386B454 GEM reference to evaluate the iAp386B454 GEM reaction presence or absence in 11 high-quality *Acetobacter* genomes. The most frequent missing reactions in the central metabolism, which has been published before for *A. pasteurianus* 386B (Illeghems et al., 2013; Pelicaen et al., 2019), were the ones catalyzed by proton-translocating NAD(P)^+^ transhydrogenase (EC 7.1.1.1; missing in *A. aceti* TMW2.1153, *A. tropicalis* BDGP1, *A. pasteurianus* Ab3, and *A. senegalensis* 108B), phosphoglucomutase (EC 5.4.2.2; missing in *A. aceti* TMW2.1153, *A. tropicalis* BDGP1, *A. pasteurianus* Ab3, and *A. senegalensis* 108B), ornithine cyclodeaminase (EC 4.3.1.12; missing in *A. persici* TMW2.1084, *A. aceti* TMW2.1153, and *A. ghanensis* LMG 23848^T^), a putative periplasmic mannitol oxidation reaction (missing in *A. ascendens* LMG 1590^T^, *A. aceti* TMW2.1153, and *A. pasteurianus* Ab3), ribose 5-phosphate isomerase (EC 5.3.1.6; missing in *A. persici* TMW2.1084, *A. tropicalis* BDGP1, and *A. senegalensis* 108B), and fructokinase (EC 2.7.1.4; missing in *A. ascendens* LMG 1590^T^ and *A. ghanensis* LMG 23848^T^). Both phosphate acetyltransferase (EC 2.3.1.8) and acetate kinase (EC 2.7.2.1) were missing in *A. aceti* TMW2.1153.

Although the lactate:H^+^ symporter was missing in *A. aceti* TMW2.1153, the genome contained genes encoding a quinone-dependent (EC 1.1.5.12) and cytochrome *c*-dependent (EC 1.1.2.4) *D*-lactate dehydrogenase. This strain was originally isolated from a water kefir and other *A. aceti* strains have been typically found in an ethanol-rich habitat, examples including vinegar. The absence of the lactate:H^+^ symporter in the *A. aceti* TMW2.1153 genome might indicate genome evolution toward excluding lactate as a substrate, other means of lactate transport in the cell, or, possibly, the erroneous prediction of absence of a gene encoding this function. In contrast, characteristic reactions for the *Acetobacter* genus, including the membrane-bound pyrroloquinoline quinone (PQQ)-dependent ethanol dehydrogenase (EC 1.1.5.5) and succinyl-CoA:acetate CoA-transferase (EC 2.8.3.18) were found in all high-quality *Acetobacter* genomes considered.

Combining the predicted orthogroups and the iAp386B454 GEM allowed to reconstruct GEMs for *A. ghanensis* LMG 23848^T^ and *A. senegalensis* 108B. GEM compositions before and after curation-based gap filling are shown in Table 3. The concomitant increase of the number of reactions and decrease of the number of dead-end metabolites indicate a successful gap filling process. A complication arose in this analysis due to the occurrence of twelve *A. pasteurianus* 386B gene identifiers in the iAp386B454 GEM, which were not present in the *A. pasteurianus* 386B NCBI RefSeq genome version used for the OrthoFinder analysis. The 10 reactions associated to these genes were computationally filtered and manually checked to see whether the presumed orthologs were present in the *A. ghanensis* LMG 23848^T^ and *A. senegalensis* 108B genomes, and if so, the reactions and associated gene-reaction rules were manually added to their respective GEMs. This was the case for D-glucose:ubiquinone oxidoreductase (EC 1.1.5.2), phosphoenolpyruvate carboxylase (EC 4.1.1.31), and 3-isopropylmalate dehydratase (EC 4.2.1.33), both for *A. ghanensis* LMG 23848^T^ and *A. senegalensis* 108B.

**Table 3:** Composition of genome-scale metabolic models (GEMs) of *Acetobacter ghanensis* LMG 23848^T^ and *Acetobacter senegalensis* 108B after automated reconstruction and curation-based gap filling.

GEM property	*A. ghanensis* LMG 23848^T^		*A. senegalensis* 108B	
	After recon-struction	After gap filling	After recon-struction	After gap filling
Compartments	2	2	2	2
Genes	306	326	339	361
Reactions	294	312	308	326
Exchange reactions	17 (6%)	17 (6%)	17 (6%)	18 (6%)
Irreversible reactions	121 (41%)	121 (41%)	132 (43%)	139 (43%)
Orphan reactions	37 (13%)	37 (13%)	37 (13%)	38 (12.0%)
Metabolites	294	297	295	298
Dead-end metabolites	26 (9%)	9 (3%)	13 (4%)	4 (1%)

For *A. ghanensis* LMG 23848^T^, a quinone-dependent *D*-lactate dehydrogenase (EC 1.1.5.12) was absent in the genome, but a cytochrome *c*-dependent enzyme (EC 1.1.2.4) was identified. No acetolactate decarboxylase (EC 4.1.1.5) was found in the *A. ghanensis* LMG 23848^T^ genome. Instead, a gene encoding diacetyl reductase (EC 1.1.1.303) was present. Finally, no quinone-dependent glycerol 3-phosphate dehydrogenase (EC 1.1.5.3) was found. Whereas for *A. senegalensis* 108B no proton-translocating NAD(P)^+^ transhydrogenase (EC 7.1.1.1) was found in the genome, a soluble NAD(P)^+^ transhydrogenase (EC 1.6.1.1) was present. Additionally, no gene could be found encoding gluconokinase (EC 2.7.1.12), possibly explaining why *A. senegalensis* 108B could not grow under defined medium conditions with glucose as the sole carbon source. The fact that citrate could sustain growth of this strain paved the way to find a genomic cause. Indeed, a putative citrate:H^+^ symporter and a gene cluster encoding citrate lyase was found in the genome, as previously reported (Illeghems et al., 2016). The gene cluster encoding citrate lyase was also present in two *Acetobacter* species isolated from fruit or fruit-derived fermented foods, namely *A. persici* JCM 25330^T^ (peach fruit) and *A. malorum* CECT 7742 (strawberry vinegar). Finally, the *A. senegalensis* 108B genome encoded a phosphoenolpyruvate carboxykinase (EC 4.1.1.49), which was not found in *A. pasteurianus* 386B, thus providing another anaplerotic link between the TCA cycle and the gluconeogenesis pathway in *A. senegalensis* 108B.

### Gap filling of genome-scale metabolic models and flux balance analysis

The results of the growth experiments for *A. ghanensis* LMG 23848^T^ and *A. senegalensis* 108B were used to fill the reaction gaps in these models using the iAp386B454 GEM as a reaction repository. The FBA gap filling results indicated that 17 iAp386B454 GEM reactions were needed to obtain *in silico* growth of *A. ghanensis* LMG 23848^T^ with glucose as the sole carbon source. For *A. senegalensis* 108B, 10 iAp386B454 GEM reactions were necessary when growth was required with lactic acid as the sole carbon source. Manual curation allowed the addition of all but two reactions for *A. ghanensis* LMG 23848^T^, for which no genomic evidence could be found. These reactions were the ones catalyzed by asparagine synthase (EC 6.3.5.4) and ornithine cyclodeaminase (EC 4.3.1.12). Both are involved in amino acid biosynthesis, the first being necessary for *L*-asparagine biosynthesis and the latter responsible for *L*-proline biosynthesis. The gene encoding the alternative *L*-proline biosynthesis enzyme NAD(P)-dependent pyrroline-5-carboxylate reductase (EC 1.5.1.2) was annotated with an internal stop codon in the NCBI RefSeq annotation. Other biosynthesis pathways for these amino acids may exist in *A. ghanensis* LMG 23848^T^, since *in vitro* growth was obtained under defined medium conditions without the addition of amino acids. Conversely, for *A. senegalensis* 108B, genomic evidence was found for all gap filling results. However, this evidence had to be retrieved from genes in the NCBI RefSeq annotation, for which no amino acid sequence was available (e.g., due to a predicted frameshift) or genes only annotated in the GenDB or PATRIC genome annotation versions.

Flux balance analysis with the iAg23848 GEM on glucose as the sole carbon source did not result in growth, unless *L*-proline and *L*-asparagine were additionally available, in which case the predicted specific growth rate was 0.70 h^−1^ and the consumption of both amino acids was balanced by the protein biosynthesis reaction. The flux distribution revealed obligatory NADPH oxidation by the proton-translocating NAD(P)^+^ transhydrogenase. However, genome annotation analysis did not support the presence of this reaction, since the *A. ghanensis* LMG 23848^T^ genome missed the 𝛽-subunit of this enzyme. Growth of *A. ghanensis* LMG 23848^T^ under defined medium conditions with glucose was only found when a citrate buffer was used. Allowing additional *in silico* consumption of citrate showed that constraining the proton-translocating NAD(P)^+^ transhydrogenase did not prevent growth, resulting in the prediction of a specific growth rate of 0.58 h^−1^ with the glutamate synthase reaction (EC 1.4.1.13) as the largest NADPH consumer.

The iAs108B GEM was solved with FBA under two growth conditions, namely with lactic acid or with citric acid as the sole carbon sources. No additional nutrients had to be added to obtain *in silico* growth, suggesting that this model contained all reactions necessary for biomass formation. FBA with lactic acid resulted in a specific growth rate of 0.86 h^−1^. The soluble NAD(P)^+^ transhydrogenase produced NADPH, whereas phosphoenolpyruvate carboxykinase supplied all necessary phosphoenolpyruvate. FBA with citric acid resulted in a specific growth rate of 0.75 h^−1^. Most of the citric acid consumed was directly used in the TCA cycle, whereas only 12% was consumed by citrate lyase. No acetate was produced by the model; all intracellular acetate produced was consumed by succinyl-CoA:acetate CoA-transferase.

### Presence of the glyoxylate cycle and aerobic respiratory chain reactions

The presence of genes encoding the glyoxylate cycle enzymes was assessed in all 36 *Acetobacter* genomes examined. Presence/absence was compared to experimental data related to the growth of the strains in a defined medium containing ethanol as the sole carbon source and obtained for the type strains of a number of *Acetobacter* species (Table 4; Cleenwerck et al., 2008). For strains of eight out of 19 *Acetobacter* species that were able to grow, three species (*A. estunensis*, *A. peroxydans*, and *A. lovaniensis*) had at the time of analysis no genome sequenced, two species for which a genome sequence was available (*A. aceti* and *A. nitrogenifigens*) encoded the glyoxylate cycle, and finally, three species with a genome sequence available (*A. senegalensis*, *A. cibinongensis*, and *A. fabarum*) did not encode the glyoxylate cycle. The latter three species could thus correspond to false positive experimental results based on growth with ethanol as the sole carbon source that were obtained formerly (Table 4) and should hence be repeated, albeit that the genome sequence available for *A. fabarum* is not that from the type strain.

**Table 4:** Overview of the presence of the glyoxylate cycle enzymes in genomes of strains of *Acetobacter* species.

*Acetobacter* species (number of strains tested in Cleenwerck et al., 2008)	Growth on ammonium + ethanol (according to Cleenwerck et al., 2008)	Number of genomes included in the OrthoFinder analysis	Presence of a gene encoding isocitrate lyase (EC 4.1.3.1)	Presence of a gene encoding malate synthase (EC 2.3.3.9)
*A. fabarum* (4)	+	1	No	No
*A. lovaniensis* (1)	+	0	?	?
*A. ghanensis* (3)	−	1	No	No
*A. syzygii* (1)	−	0	?	?
*A. pasteurianus* (7)	−	3	No	No
*A. pomorum* (1)	−	1	No	No
*A. peroxydans* (2)	+	0	?	?
*A. indonesiensis* (2)	−	1	No	No
*A. orientalis* (1)	−	1	No	No
*A. cibinongensis* (1)	w	1	No	No
*A. tropicalis* (2)	−	2	No	No
*A. senegalensis* (3)	+	2	No	No
*A. orleanensis* (4)	−	2	No	No
*A. malorum* (1)	−	2	No	No
*A. cerevisiae* (4)	−	2	No	No
*A. nitrogenifigens* (1)	+	1	Yes	Yes
*A. oeni* (1)	−	0	?	?
*A. aceti* (4)	+	2	Yes	Yes
*A. estunensis* (3)	+	0	?	?

Growth, +; no growth, −; weak growth, w. If the results of only one strain are reported, this is always the type strain.

?, no genome available at the time of investigation.

The enzymes involved in the aerobic respiratory chain as known for *E. coli* were present in all *Acetobacter* genomes considered, comprising NADH dehydrogenase type I (EC 7.1.1.2), NADH dehydrogenase type II (EC 1.6.5.9), cytochrome *bo_3_* oxidase (EC 7.1.1.3), and cytochrome *bd* oxidase (EC 7.1.1.7). Concerning NADPH metabolism, *E. coli* contains both a proton-translocating NAD(P)^+^ transhydrogenase and a soluble NAD(P)^+^ transhydrogenase. From the comparative genomic analysis, it was apparent that, at least for the high-quality *Acetobacter* genomes, the presence of these enzymes was mutually exclusive. An interesting exception was *A. aceti*, for which both reactions were predicted to be present.

## Discussion

The reconstruction of a GEM for *A. ghanensis* LMG 23848^T^ and *A. senegalensis* 108B, two candidate starter culture strains for cocoa fermentation (Illeghems et al., 2016), provided *in silico* tools to gain insights into the metabolic properties of these strains. In the current study, the GEM reconstruction process was accelerated using a semi-automated approach with experimental support. Growth experiments under defined medium conditions identified individual carbon sources that allowed the growth of *A. ghanensis* LMG 23848^T^ and *A. senegalensis* 108B. These growth conditions could then be used in simulations using FBA with the newly reconstructed iAg23848 and iAs108B GEMs, providing insights into the metabolic flux distributions of the models.

For *A. senegalensis* 108B, the prediction of citrate assimilation based on a previous genome analysis (Illeghems et al., 2016) was confirmed. In general, conversion of citrate present in the cocoa pulp-bean mass is ascribed to lactic acid bacteria that proliferate at the start of a cocoa fermentation process (De Vuyst and Weckx, 2016; De Vuyst and Leroy, 2020). Citrate consumption by AAB species may contribute to their survival during this more or less anaerobic stage of a cocoa fermentation process (Illeghems et al., 2016). For *A. ghanensis* LMG 23848^T^, the absence of a quinone-dependent *D*-lactate dehydrogenase (EC 1.1.5.12) was probably compensated by the presence of a cytochrome *c*-dependent enzyme (EC 1.1.2.4), which probably allowed lactate consumption. However, this may be an explanation for the lower lactate consumption rate of *A. ghanensis* LMG 23848^T^, compared to *A. pasteurianus* 386B, under cocoa pulp-simulating conditions (Moens et al., 2014). In general, both lactate and acetate are overoxidized during a late stage of a cocoa fermentation process (Moens et al., 2014; De Vuyst and Weckx, 2016; De Vuyst and Leroy, 2020). Additionally, no acetolactate decarboxylase (EC 4.1.1.5) was found in the *A. ghanensis* LMG 23848^T^ genome, although acetoin was formed (Moens et al., 2014). In *A. pasteurianus*, it has been shown that acetoin production solely depends on acetolactate decarboxylase, in contrast to the theoretically proposed acetaldehyde carboligation pathway, as found in *Saccharomyces cerevisiae* (De Ley, 1959; Gunawan et al., 2007). However, in *A. ghanensis* LMG 23848^T^ as well as in *A. senegalensis* 108B, yet another pathway may be responsible for acetoin formation, since in both genomes a gene encoding diacetyl reductase (EC 1.1.1.303) was present, as is the case in lactic acid bacteria (Keenan and Lindsay, 1968). Finally, no quinone-dependent glycerol 3-phosphate dehydrogenase (EC 1.1.5.3) could be found, possibly explaining why *A. ghanensis* LMG 23848^T^ could not grow under defined medium conditions with glycerol as the sole carbon source.

Re-annotation of the genomes of *A. ghanensis* LMG 23848^T^ and *A. senegalensis* 108B proved to be critical to reconstruct their GEMs. Reaction presence predictions using the iAp386B454 GEM as a reference were successful, but led to metabolic network gaps in essential biosynthesis reactions. Without the current re-annotation effort, it would be impossible to speculate about their presence. Keeping this in mind, the mutual occurrence of genome sequence errors in query and reference genomes blurs the outcome of this functional comparative genomic analysis, since it is ultimately based on the prediction of the presence or absence of genes in the genomes considered. However, considering that genomic data are the only well-structured and publicly available data source at hand to compare the metabolic properties of *Acetobacter* species, this analysis may provide interesting insights for future research. The predicted differences in the carbon and redox metabolic potential of the *Acetobacter* species investigated revealed the possible metabolic diversity in this genus. Specifically, the presence or absence of the lactate:H^+^ symporter, fructokinase, gluconokinase, ribose 5-phosphate isomerase, and citrate lyase may represent adaptations to different habitats and niches. Also, the presence or absence of a proton-translocating and soluble NAD(P)^+^ transhydrogenase may represent an important and hitherto not sufficiently explored adaptation to the use of different carbon sources and their influence on the redox metabolism of *Acetobacter* species (Pelicaen et al., 2020). In *E. coli*, both enzymes are present, each having a specific functional role, whereby the first enzyme produces NADPH and the latter re-oxidizes excess NADPH (Sauer et al., 2004). The complete coverage of the key reactions of the aerobic respiratory chain among the *Acetobacter* genomes is a strong indication that this metabolic feature is a defining physiological factor of this genus.

The predicted presence (e.g., *A. aceti* and *A. nitrogenifigens*) or absence (e.g., *A. ghanensis*, *A. senegalensis*, and *A. pasteurianus*) of the glyoxylate cycle enzymes represented an interesting case where genomic and experimental evidence go hand in hand. Alternative pathways have been described for the assimilation of C2 compounds in microorganisms, namely the ethylmalonyl-CoA pathway and the methylaspartate cycle (Alber et al., 2006; Khomyakova et al., 2011). However, these pathways have not been found in AAB, and no genomic evidence for their presence could be found in the *Acetobacter* genomes considered. If the glyoxylate cycle is the only C2 assimilation pathway present in species of the genus *Acetobacter*, then the phenotypic test concerning the growth of an *Acetobacter* species in a medium with ethanol as the sole carbon source, commonly used to infer the taxonomy of an unknown *Acetobacter* strain, should be backed by genomic evidence. The results presented here indicate that this was indeed not the case for some *Acetobacter* species, thus questioning experimental data (Cleenwerck et al., 2008). *Acetobacter senegalensis* 108B was not able to grow under defined medium conditions with ethanol as the sole carbon source. In addition, no genomic evidence was found for the glyoxylate cycle in the re-annotated *A. senegalensis* 108B genome. The experimental results presented here are in contrast to previous results obtained for *A. senegalensis* 108B and for the type strain of *A. senegalensis* (Camu et al., 2007; Ndoye et al., 2007). Thus, this is an argument that genomic evidence should be taken into account for future species descriptions of members of the *Acetobacter* genus.

From a practical viewpoint, in fermentation processes such as vinegar production, the use of strains that can solely oxidize ethanol to acetic acid, but not assimilate it into biomass components, may be favorable (Mullins and Kappock, 2013; Sakurai et al., 2013). Similarly, for the fermentation of cocoa pulp-bean mass, the absence of the glyoxylate cycle in the genomes of *A. ghanensis* LMG 23848^T^, *A. senegalensis* 108B, and *A. pasteurianus* 386B (Illeghems et al., 2013; Pelicaen et al., 2019), may represent a competitive advantage of these strains to quickly oxidize the available ethanol, produced by yeast species, to acetic acid. Genomic screening of strains may provide a more straightforward strategy to select those strains for which no evidence of the glyoxylate cycle can be found, to use them as starter cultures in such fermentation processes.

## Conclusion

In the present study, the possibility of integrating two different data structures based on the same genomic data source was explored. A first data structure comprises GEMs, allowing to catalogue the entire biochemical reaction potential of a microbial strain and *in silico* metabolic flux simulations. Also, gene-reaction rules, manually curated or not, allow to explore the causal links between the genome and the reactome. A second data structure, the orthogroups, predicts the phylogenetic relatedness between protein-encoding genes from different species genomes, from which estimates of their shared function can be made. Combining the manually curated gene-reaction rules of a GEM of *A. pasteurianus* 386B and *E. coli* and a set of predicted orthogroups of a selection of *Acetobacter* genomes allowed to predict the presence of reactions that characterized the metabolism of *A. pasteurianus* 386B and *E. coli* for these *Acetobacter* species. Evaluation of biochemical reaction presence in bacterial genomes represents a new frontier in genome annotation, since this evaluation is a step closer to the actual biological functionalities that the genome encodes for. The results obtained stress the need for proper data curation and species description.

## Data availability statement

The datasets presented in this study can be found in online repositories. The genome-scale metabolic models in xml format can be found at https://zenodo.org under submission number 6320681 or under DOI at https://doi.org/10.5281/zenodo.6320681.

## Author contributions

RP carried out the research and drafted the manuscript. DG, SW, and LDV supervised the research and revised and edited the manuscript. All authors approved the manuscript.

## Funding

The authors acknowledge financial support from the Research Council of the Vrije Universiteit Brussel (SRP7 and IOF342 projects). RP was the recipient of a PhD Fellowship strategic basic research of the Research Foundation Flanders (FWO-Vlaanderen; 1S27316N).

## Conflict of interest

The authors declare that the research was conducted in the absence of any commercial or financial relationships that could be construed as a potential conflict of interest.

## Publisher’s note

All claims expressed in this article are solely those of the authors and do not necessarily represent those of their affiliated organizations, or those of the publisher, the editors and the reviewers. Any product that may be evaluated in this article, or claim that may be made by its manufacturer, is not guaranteed or endorsed by the publisher.
